# Effort–Reward Imbalance at Work and Prescription Drug Misuse—Prospective Evidence from Germany

**DOI:** 10.3390/ijerph19137632

**Published:** 2022-06-22

**Authors:** Sebastian Sattler, Olaf von dem Knesebeck

**Affiliations:** 1 Faculty of Sociology, Bielefeld University, Universitaetsstrasse 25, 33615 Bielefeld, Germany; 2 Institute for Sociology and Social Psychology, University of Cologne, Universitaetsstrasse 24, 50931 Cologne, Germany; 3 Pragmatic Health Ethics Research Unit, Institut de Recherches Cliniques de Montréal (IRCM), 110, Avenue des Pins Ouest, Montréal, QC H2W 1R7, Canada; 4 Institute of Medical Sociology, University Medical Center Hamburg-Eppendorf, Martinistrasse 52, 20246 Hamburg, Germany; o.knesebeck@uke.de

**Keywords:** prescription drug misuse, cognitive enhancement, effort–reward imbalance, stress, overcommitment

## Abstract

This study examines how work stress affects the misuse of prescription drugs to augment mental performance without medical necessity (i.e., cognitive enhancement). Based on the effort–reward imbalance model, it can be assumed that a misalignment of effort exerted and rewards received increases prescription drug misuse, especially if employees overcommit. To test these assumptions, we conducted a prospective study using a nationwide web-based sample of the working population in Germany (*N* = 11,197). Effort, reward, and overcommitment were measured at *t*_1_ and the 12 month frequency of prescription drug misuse for enhancing cognitive performance was measured at a one-year follow-up (*t*_2_). The results show that 2.6% of the respondents engaged in such drug misuse, of which 22.7% reported frequent misuse. While we found no overall association between misuse frequency and effort, reward, or their imbalance, overcommitment was significantly associated with a higher misuse frequency. Moreover, at low levels of overcommitment, more effort and an effort–reward imbalance discouraged future prescription drug misuse, while higher overcommitment, more effort, and an imbalance increased it. These findings suggest that a stressful work environment is a risk factor for health-endangering behavior, and thereby underlines the importance of identifying groups at risk of misusing drugs.

## 1. Introduction

Stress concepts addressing the social environment and the consequences for exposed people often focus on work and employment. The effort–reward imbalance (ERI) model was established for examining the consequences of work stress [1,2,3], with roots in the biopsychosocial stress theory [4,5,6]. The model concentrates on the experienced lack of social reciprocity. Accordingly, an imbalance between high efforts spent and low rewards (such as esteem, salary/job promotion, or job security) can elicit negative emotions and harmful stress. The model also consists of an intrinsic component (overcommitment), which defines a motivational pattern of excessive work-related commitment and a high need for approval. Overcommitment can amplify an effort–reward imbalance or independently evoke emotional stress. Numerous studies have shown the adverse health effects of an imbalance between effort and reward, especially with regard to cardiovascular diseases [7], affective disorders [8], immune function [9], and suicidal ideation [10]. There is also some evidence for associations between ERI and health damaging behaviors, like tobacco use [11] and heavy alcohol consumption [12,13]. However, not all studies have found higher behavioral risks among people experiencing a high effort and low reward at work [11,14].

Some studies also analyzed the relationship between psychosocial stress at work and drug misuse. For example, Choi [15] found psychosocial work stressors to be associated with opioid use disorder in US workers, while another US study conducted by Wiesner et al. [16] did not find a direct relationship between job stress and drug use. In terms of the ERI model, a French study revealed a significantly increased risk for long-term benzodiazepine use among workers suffering from ERI [17]. Another study that cross-sectionally examined associations with misusing multiple drugs in a national sample of US workers [18] found significantly higher odds of misusing any drugs among individuals experiencing high effort and low reward at work, but not for all indicators of drug misuse. The authors concluded that further prospective evidence is needed and suggested examining the role of overcommitment as an important vulnerability factor for a more complete test of the model. They also mentioned the problem that many studies cannot clearly distinguish between medically prescribed drugs and drug misuse. These studies only focus on prescribed use, while drug misuse warrants its own focus (e.g., due to assessing contaminated drugs on illegal markets or no medical supervision).

Misusing drugs that are prescribed to treat diseases such as attention-deficit hyper-activity disorder or narcolepsy is a way to deal with cognitive requirements and strain [19,20,21,22,23,24]. These drugs include substances such as modafinil (e.g., Provigil), methylphenidate (e.g., Ritalin), or amphetamine−dextroamphetamine (e.g., Adderall). Individuals also misuse them without a prescription to counteract sleepiness and exhaustion or to improve their memory and concentration, i.e., with the intention of retaining or improving their cognitive performance. Some studies suggest that the 12 month prevalence of cognitive enhancement with prescription drugs in Germany, the country in which we conducted our study, increased from 1.5% in 2015 to 3.0% in 2017. However, this was reported in a study using a self-selected population [25]. Representative studies are rare in this context, e.g., [26,27] and several (often non-representative) studies focus on specific occupations, such as scientists [28], doctors, programmers, advertising specialists, and publicists [29]. The misuse of such drugs for enhancement purposes has been positively described as a means to increase productivity and wealth [30,31]. However, there is also concern given the possible negative health consequences arising from such drug misuse, which can include addiction. Moreover, such use may result in drug-related crimes or pressure on non-users to take drugs to compete with drug users and increase performance standards [32,33,34]. Therefore, investigating the drivers of such drug misuse is important for public health interventions, occupational health, and policy making.

Although an association between work stress and the misuse of such prescription drugs to augment mental performance is reasonable as it may help workers cope with acute stressful situations, demands of the job, and the consequences of sustained stress as a maladaptive coping strategy [19,27,35,36,37], empirical evidence is limited to cross-sectional research. Moreover, we know of no study investigating the role of ERI or overcommitment on such drug misuse, nor how overcommitment may shape the possible effects of ERI. Against this background, the purpose of this study is to prospectively explore the relationship between prescription drug misuse for cognitive enhancement and ERI, its two dimensions (effort and reward), and overcommitment and its conditioning effect on ERI.

## 2. Materials and Methods

### 2.1. Participants

To examine our assumptions, we conducted a web-based study (ENHANCE) with an offline-recruited nationwide sample of adult (18 or older) residents in Germany (who have internet access, which applies to about 95% of all households [38]). The sample at *t*_1_ consisted of 47,406 invited individuals, of which 27,149 (57.3%) consented to participate, and 24,809 (91.4%) completed the study. The sample combines an initial sample of 37,003 invited individuals (with 24,085 consenting individuals and 22,024 completers) and an extended sample of 10,403 invited individuals (with 3064 consenting individuals and 2785 completers) to counteract demographic imbalances (e.g., because of selective survey take-up by more difficult-to-reach participants) and increase the quality of the sample. One year later, 24,683 individuals (adjusted for those who dropped out of the survey, died, etc.) were invited, of which 17,818 (72.2%) consented to participate, and 15,235 (85.5%) completed the survey. Participants who completed both surveys received bonus points as motivational incentives (approximately $5.30), which they could convert into vouchers, a ticket for a charity lottery, or a donation to UNICEF.

Our analytical sample was restricted to 11,197 employed individuals who responded to all of the variables used in the analysis (50.0% women, mean age: 48.16 years, see Table 1). The ENHANCE study was approved by the ethics committee of the University of Erfurt (reference numbers: EV-20190917 and EV-20200805).

### 2.2. Instruments

#### 2.2.1. Dependent Variable

At *t*_2_, respondents were asked to indicate how often they had taken prescription drugs in the last 12 months to support their mental performance, without the drugs being taken on the advice of a doctor to treat an illness, c.f., [26,28]. Before completing the questions, participants were told that such medications are usually taken to treat diseases (like attention deficit disorder, narcolepsy, dementia, depression, and anxiety), and that we were interested in their misuse of prescription drugs, including stimulants (e.g., Ritalin), anti-dementia drugs (e.g., Piracetam), beta blockers (e.g., Metoprolol), antidepressants (e.g., Fluoxetine), and others. Response options were “0 times” (coded as 1), “1–2 times” (2), “3–5 times” (3), “6–9 times” (4), “10–19 times” (5), “20–39 times” (6), and “40+ times” (7), similar to other studies on drug misuse frequencies [39,40].

#### 2.2.2. Independent Variables

Effort–reward imbalance (ERI): At *t*_1_, we used the short measure of ERI at work [41], consisting of three items for “effort” (e.g., “My workload has become larger and larger.”), seven items for “reward”, two items for “esteem” (e.g., “I get the recognition I have earned from my supervisor and/or equally important person”), two items for “job security” (e.g., “My own job is at risk”, reverse coded), and three items for “job promotion” (e.g., “When I think of all of my work and effort, I think my salary/wages are appropriate”). All of the items referred to the past 12 months (please see Table A2 for the full wording of all items). We used a four-point response scale with the response options “do not agree at all” (1), “more or less disagree” (2), “more or less agree” (3), and “completely agree” (4). In addition, we did not use the two-step response process, which, according to Tsutsumi et al. [42], leads to measurement errors and places high cognitive demands on respondents. According to Siegrist et al. [43], item nonresponse can be substantially reduced by one-step measures. The scale reliability coefficients were 0.76 for effort and 0.77 for reward. To adjust for the unequal number of items, we first calculated mean scores of the three effort items and seven reward items before we calculated a weighted ratio between the scales “effort” and “reward”, i.e., the effort–reward ratio (ERR). This quantifies the degree of the individual mismatch between high “cost” and low “gain” situations. Thereby, scores above 1 indicate more effort compared with reward and scores below 1 indicate more reward than effort.

Overcommitment: We measured overcommitment during the past 12 months at *t*_1_ with four [41] out of six original items [44]. An exemplary item is “Those closest to me say that I sacrifice too much for my job”. Response options resemble those of ERI. The scale reliability coefficient was 0.81.

### 2.3. Statistical Analysis

Prospective ordinary least squares (OLS) regression models were computed with Stata 14.2 to test the effects of the predictors at *t_1_* on the logarithmic 12 month drug misuse frequency measured at *t_2_*. We used the natural logarithm [45,46] because, as frequently observed in other studies on such drug misuse and deviant or criminal behavior, e.g., [39], responses were positively skewed. Through the transformation, the variable appeared more “normally” distributed for the analysis. We first examined the main effects of effort, reward, and overcommitment (Model 1) and of the ERR and overcommitment (Model 2), followed by two models testing for the conditioning effect of overcommitment on effort and reward (Model 3), and on the ERR (Model 4). In all of our analyses, we controlled for gender, age, and a dichotomous indicator of prior lifetime prescription drug misuse for enhancement purposes at *t*_1_.

## 3. Results

We found that, on average, respondents indicated that they put more effort in their work than the rewards they received (Table 1); more than half of the respondents indicated an ERR above 1.

**Figure 1:**
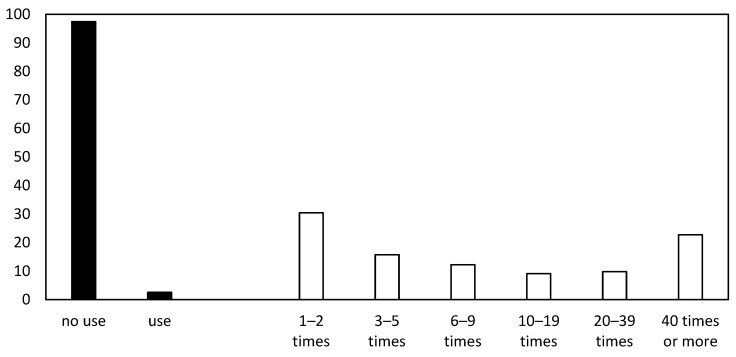
Prevalence in percent of CE-drug misuse during the past 12 months (■, at *t*_2_, *N* = 11,197) and respective misuse frequencies from those reporting such drug misuse (□, at *t*_2_, *N* = 291).

Figure 1 shows that the majority of respondents (97.4%, *n* = 10,911) reported no misuse of prescription drugs for enhancement purposes within the past 12 months and 2.6% (*n* = 286) reported a misuse. Of the latter, almost one third (30.4%, *n* = 87) used such drugs one or two times and more than one fifth (22.7%, *n* = 65) reported using prescription drugs 40 times or more in the last 12 months. Approximately 4.5% (*n* = 502) of respondents reported a misuse of prescription drugs for enhancement prior to the past 12 months (i.e., at *t*_1_). Of these 502 respondents, 399 (79.5%) reported no misuse at *t*_2_ and may have (temporally) turned to abstainers, while 103 (20.5%) reported continuous prescription drug misuse at *t*_2_. Of the 10,695 respondents not reporting any misuse at *t*_1_, 183 (1.7%) may have initiated prescription drug misuse between *t*_1_ and *t*_2_, while the majority of the 10,512 (98.3%) participants remained non-users.

Regression analyses (Table 2, Model 1) showed no statistically significant association between effort (*p* = 0.186) and reward (*p* = 0.081) and the frequency of prescription drug misuse for enhancement at *t*_2_. However, respondents with higher overcommitment at *t*_1_ reported more frequent misuse at *t*_2_ (*p* < 0.001). From the controls, only prior drug misuse was significantly associated with the outcome (*p* < 0.001). Model 2 shows that the ratio between effort and reward had no statistically significant association with prescription drug misuse frequency (*p* = 0.416).

**Figure 2:**
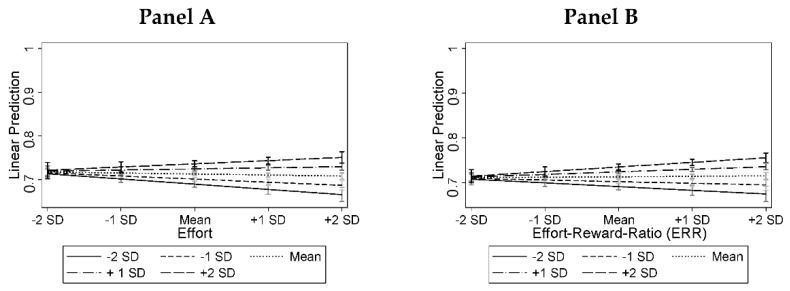
Predictive margins with 95% CIs for the 12-month CE-drug misuse frequency measured at *t*_2_ depending on effort (**Panel A**, based on model 3), the effort–reward-ratio (ERR, **Panel B**, Based on Model 4), and the conditioning role of overcommitment, which is indicated by the different plotted lines with varying ascent (all measured at *t*_1_). *Notes:* Results are plotted for different combinations of values (i.e., from minus two standard deviations to plus two standard deviations) of effort, ERR, and overcommitment.

However, we found conditional relationships between the level of overcommitment and effort (*p* = 0.002, Model 3) and ERR (*p* = 0.007, Model 4). Panel A in Figure 2 shows, for individuals with low levels of overcommitment, that higher effort is associated with a lower frequency of prescription drug misuse, but for individuals with high levels of overcommitment, exerting more effort increased this frequency. A similar pattern was found for ERR (Panel B), which might be driven by the interaction between effort and overcommitment reported before. Thus, for respondents with low levels of overcommitment, an effort–reward imbalance reduced the prospective prescription drug misuse frequency, while for individuals with high levels of overcommitment, an imbalance towards more effort than reward increased the frequency of misuse.

## 4. Discussion

### 4.1. Summary and Interpretation

In this study, associations between work stress according to the ERI model and misusing prescription drugs for cognitive enhancement were explored. The results indicated that 2.6% of 11,197 individuals employed in Germany reported a misuse of drugs in the past 12 months. While this number is in the range of previous studies in Germany [25,26,47,48], we also found that one in five users reported using drugs 40 times or more in the last 12 months. In terms of work stress, more than half of the respondents had an effort–reward ratio above 1, indicating more effort than the gained reward. Thus, compared with other prospective cohort studies [49], the prevalence of ERI is high in our sample.

Moreover, we found no overall relation of effort, reward, or the respective imbalance with prescription drug misuse for cognitive enhancement. However, people who were excessively committed to their work were prospectively using prescription drugs more frequently for enhancing their performance. Apart from this association, overcommitment conditioned the prospective effects of effort and ERR, i.e., increasing overcommitment, thus with more effort, and creating an imbalance between effort and reward that encouraged prescription drug misuse. Previous studies examining associations between psychosocial stress at work applying the ERI model and drug misuse revealed inconsistent results [17,18]. However, to the best of our knowledge, this is the first study specifically focusing on the relationship between ERI and the misuse of prescription drugs to augment mental performance.

By using the ERI model, our analyses were based on an established theoretical approach suitable for examining the consequences of work stress in a wide range of occupations and contexts [1,2,3]. The model assumes that a lack of reciprocity between effort expended at work and rewards received (such as esteem, salary/job promotion, or job security) is associated with strong negative emotions and sustained biological stress responses. This lack of reciprocity is conceptualised as an effort–reward imbalance. The model additionally includes an intrinsic component (overcommitment), which defines a motivational pattern of excessive work-related commitment and a high need for approval. Accordingly, overcommitment can amplify an effort–reward imbalance or independently evoke emotional stress. There is evidence of adverse health effects for an imbalance between effort and reward, e.g., [7,8], and some studies suggest associations with health-damaging behaviors [11,14] and drug misuse [17,18]. However, the role of overcommitment was missing in several previous studies using the ERI model in the context of drug misuse, e.g., [18].

Our findings indicate that the intrinsic component of the ERI model seems more important for this type of drug misuse than the extrinsic components representing the working situation. In a way, this is plausible, as cognitive enhancement drug use and overcommitment can both be considered critical coping styles [28,36,50]. Overcommitment is a specific pattern for coping with demanding situations, characterized by excessive engagement and a desire for being in control. Overcommitted people are more likely to continuously strive towards high achievements in their jobs because of their underlying need for approval or esteem at work [51]. This can also lead to exhaustion. Obviously, these people tend to misuse more drugs to improve their cognitive performance and productivity, possibly also as a resource for compensation.

Under the condition of overcommitment, high effort and an imbalance between effort and reward was positively associated with prescription drug misuse. In other words, the extrinsic components characterizing the work situation seem to promote drug misuse only among overcommitted people. Interestingly, people with low or a lack of overcommitment are less likely to misuse drugs when effort is high. Thus, their work motivation may not be high enough to make them willing to take the risk of experiencing side effects or face the challenge of obtaining such drugs, e.g., by feigning symptoms at the doctor’s or finding relatives or friends who may share their medication [52,53,54]. These findings also suggest that, in the absence of a main effect of work stress on the outcome (here prescription drug misuse), work stress may exert differential effects, i.e., the investigated type of drug misuse follows different patterns depending on the combination of the extrinsic and the intrinsic component of the model. This may further inform research on the differential effects of work stress depending on the respondent characteristics, as also implied in other models on occupational stress, such as the job demands-resources model [19,55,56].

### 4.2. Strengths, Limitations, and Future Research

Because previous studies on cognitive enhancement have often used cross-sectional designs with smaller scale studies that were only partially engaged with sub-populations of the working population (such as scientists or surgeons) [28,57], this study has the strength of its prospective design with a large sample based on a random selection of respondents. Thus, the design is aimed at counteracting the improper temporal ordering of measures that has been criticized in cross-sectional studies [36]. Moreover, we used a frequency measure instead of a dichotomous drug misuse measure to better capture the extent of drug misuse and not to treat one-time drug misusers like frequent misusers. Our data, therefore, may help identify misusers who might be at risk of addiction [20]. However, self-reported drug misuse measures could be subject to socially desirable responding [58]. We, therefore, ensured anonymous reporting through the fact that the researchers never had access to personal data. Moreover, additional analyses showed that the results remained stable when controlling for anonymity perceptions towards the survey [59] (Table A1).

Future studies should investigate longer periods of drug misuse than 12 months, given that, especially long-term imbalances between effort and reward, as well as overcommitment, are expected to be relevant for motivating people to misuse performance-enhancing drugs. Furthermore, it is worthwhile to consider socio-economic factors, like income or occupational status, as these may exert significant effects or moderate the effects of ERI or overcommitment. For a better understanding of the association between ERI, overcommitment, and prescription drug misuse, future research with more waves of data collection might examine the mediating effect of work motivation, emotional stress, or the depletion of cognitive energy, which may result from the imbalance between effort, reward, and overcommitment.

## 5. Conclusions

Our results suggest using prescription drugs to increase cognitive functioning without medical indication is not as prevalent as the media sometimes depicts [60,61,62]. Still, when extrapolating our prevalence rates to the working population, approximately 1.8 million individuals in Germany misused prescription drugs for such purposes within a year. Of these, about 400,000 individuals misused such drugs relatively frequently. Frequent misuse can cause health-related side effects, such as nausea, hypertension, sleep problems, or addiction, the chances of which might be underestimated because of an optimism bias [63,64,65], and they could be particularly likely when such drugs are bought on the black market. Moreover, if peers and colleagues learn about the prescription drug misuse of an individual, this can lead to effects of contagion and indirect coercion to also misuse such drugs in order to keep up. There is also evidence supporting this conjecture [66,67,68]. While such effects have led researchers to expect cognitive enhancement to become more widespread [69,70], our results suggest that a high motivational pattern of excessive work-related commitment may further maintain or increase the misuse of prescription drugs for enhancement, as prescription drugs seem to be seen as an instrumental resource to achieve high performance and to manage this stress [19,28,71]. This might be especially likely if other resources such as social support are lacking. From our finding, we can also conclude that, in particular, overcommitted individuals who are under stress due to high chronic work effort (e.g., through time pressure, a high workload, or disruption) are at an especially elevated risk for searching for pharmaceutical means to manage their demands and cope with stress. This is important to reflect upon given the continuous stress-inducing transformations in the work environment, such as digitalization and flexibilization [2,71,72,73]. Individuals who are exposed to continuous work stress with a high willingness to overexert themselves may ignore or downplay the signals from their body and mind and may engage in health-endangering behavior, such as drug misuse. These individuals especially should be targeted for prevention and intervention programs. Tools for stress management, such as relaxation or meditation, could be particularly helpful, while structural changes of adverse work environments also need to be made [74].

## Figures and Tables

**Table 1:** Descriptive Information (*N* = 11,197) ^a^.

	*Mean*	*SD*	*Min*	*Max*
12 month CE-drug misuse at *t*_2_	1.08	0.595	1.00	7.00
12 month CE-drug misuse at *t*_2_ (Log)	0.72	0.152	0.69	2.08
Effort	2.79	0.716	1.00	4.00
Reward	2.69	0.561	1.00	4.00
Effort–reward ratio (ERR)	1.11	0.459	0.25	4.00
Overcommitment	2.35	0.735	1.00	4.00
Prior CE-drug misuse (Dummy)	0.04	0.207	0.00	1.00
Female	0.50	0.500	0.00	1.00
Age	48.16	11.590	18.00	86.00

*SD* = standard deviation; *Min* = minimum, *Max* = maximum. ^a^ Please see Methods section for response scales of each construct.

**Table 2:** Logarithmic 12 month misuse frequency of CE drugs (measured at *t*_2_), based on ordinary least squares regression models (*N* = 11,197).

	Model 1	Model 2	Model 3	Model 4
Effort	−0.003		−0.022 ***	
	[−0.007, 0.001]		[−0.035, −0.009]	
Reward	−0.005		−0.005	
	[−0.010, 0.001]		[−0.022, 0.012]	
Effort–reward ratio (ERR)		0.003		−0.024 *
		[−0.004, 0.010]		[−0.044, −0.003]
Overcommitment	0.012 ***	0.011 ***	−0.012	−0.000
	[0.008, 0.017]	[0.007, 0.015]	[−0.039, 0.015]	[−0.009, 0.009]
Effort × Overcommitment			0.008 **	
			[0.003, 0.014]	
Reward × Overcommitment			0.000	
			[−0.006, 0.007]	
ERR × Overcommitment				0.010 **
				[0.003, 0176]
Prior CE drug misuse	0.165 ***	0.165 ***	0.164 ***	0.165 ***
	[0.151, 0.178]	[0.152, 0.179]	[0.151, 0.178]	[0.151, 0.178]
Female	0.000	0.000	−0.000	0.000
	[−0.005, 0.006]	[−0.005, 0.006]	[−0.006, 0.005]	[−0.005, 0.006]
Age	0.000	0.000	0.000	0.000
	[−0.000, 0.000]	[−0.000, 0.000]	[−0.000, 0.000]	[−0.000, 0.000]
Constant	0.694 ***	0.672 ***	0.748 ***	0.700 ***
	[0.669, 0.719]	[0.657, 0.687]	[0.681, 0.815]	[0.675, 0.725]
*F-Test*	112.26 ***	134.00 ***	85.59 ***	112.95 ***

95% confidence intervals in brackets; * *p* < 0.05, ** *p* < 0.01, *** *p* < 0.001.

## Data Availability

The data that support the findings of this study are available in the repository “PUB at Bielefeld University” [75].

## Appendix A

**Table A1:** Logarithmic 12-month misuse frequency of CE drugs (measured at *t*_2_), based on ordinary least squares regression models—each model controls for anonymity perceptions (*N* = 10,853 ^a^).

	Model 1	Model 2	Model 3	Model 4
Effort	−0.004		−0.021 **	
	[−0.009, 0.000]		[−0.034, −0.008]	
Reward	−0.004		−0.003	
	[−0.010, 0.001]		[−0.020, 0.014]	
Effort–reward ratio (ERR)		0.002		−0.026 *
		[−0.005, 0.009]		[−0.046, −0.005]
Overcommitment	0.012 ***	0.011 ***	−0.007	−0.001
	[0.008, 0.017]	[0.006, 0.015]	[−0.034, 0.020]	[−0.010, 0.008]
Effort × Overcommitment			0.007 **	
			[0.002, 0.013]	
Reward × Overcommitment			−0.001	
			[−0.007, 0.006]	
ERR × Overcommitment				0.011 **
				[0.003, 0.018]
Prior CE drug misuse	0.160 ***	0.161 ***	0.159 ***	0.160 ***
	[0.147, 0.173]	[0.147, 0.174]	[0.146, 0.173]	[0.146, 0.173]
Female	0.000	0.000	−0.000	0.000
	[−0.005, 0.006]	[−0.005, 0.006]	[−0.005, 0.005]	[−0.005, 0.006]
Age	0.000	0.000	0.000	0.000
	[−0.000, 0.000]	[−0.000, 0.000]	[−0.000, 0.000]	[−0.000, 0.000]
Anonymity perceptions	−0.010 ***	−0.010 ***	−0.010 ***	−0.010 ***
	[−0.013, −0.006]	[−0.013, −0.006]	[−0.013, −0.006]	[−0.014, −0.006]
Constant	0.742 ***	0.719 ***	0.784 ***	0.748 ***
	[0.713, 0.771]	[0.696, 0.741]	[0.715, 0.853]	[0.718, 0.779]
*F-Test*	95.190 ***	110.107 ***	75.067 ***	95.565 ***

95%-confidence intervals in brackets; * *p* < 0.05, ** *p* < 0.01, *** *p* < 0.001. ^a^ Lower case-number because anonymity perceptions were assessed later in the questionnaire.

**Table A2:** Effort, reward, and overcommitment items (translated from German to English).

Dimension and Sub-dimension	Items
*Effort*	
Work place	Due to the high volume of work, there has often been a lot of time pressure.
Interruptions	I have often been interrupted and disturbed during my work.
Work quantity	My workload has become larger and larger.
*Reward*	
Esteem	I get the recognition I have earned from my supervisor and/or equally important person.
	The chances of getting a promotion in my field are poor.
Job security	I am experiencing—or am expecting—my job situation to get worse.
	My own job is at risk.
Job promotion	When I think of all of my work and effort, I think I have received appropriate recognition.
	When I think of all of my work and effort, I think my personal chances for professional advancement are appropriate.
*Overcommitment*	
	It often happens to me that I’m thinking about work problems as soon as I wake up.
	Those closest to me say that I sacrifice too much for my job.
	I rarely escape work completely, it is still in my head in the evenings.
	If I postpone something which I actually should have done today, I cannot sleep at night.

Response options for all items were “do not agree at all” (1), “more or less disagree” (2), “more or less agree” (3), and “completely agree” (4).
